# A Pilot Study of the Effects of Formal Service on Japanese Family Caregivers’ Daily Fluctuation of Stress

**DOI:** 10.1093/geroni/igaa057.1144

**Published:** 2020-12-16

**Authors:** Koji Abe

**Affiliations:** Kagoshima University, Kagoshima-shi, Kagoshima, Japan

## Abstract

Purpose: Recent studies report daily fluctuations in stress among family caregivers of older individuals with dementia. Several studies focused on daily stressors or behavioral and psychological symptoms of dementia and use of adult day services. Most previous studies on daily fluctuations of caregivers’ stress have used a daily diary approach. This approach involves creating multiple daily reports, making it possible to examine between-person differences and within-person processes of change. However, only few studies used this approach for family caregivers in Asian countries. Therefore, this study examines the applicability of a daily diary approach for Japanese family caregivers and the effects of formal care services on their stress and depression. Methods: Participants were 13 family caregivers of individuals with dementia using formal care services in a rural area in Japan. They were assessed through self-administered questionnaires including use or nonuse of formal care services, caregiving stressors (DASC-8), depressive symptoms (K-6), and caregiving stress for 7 days. Generalized linear mixed models (GLMM) with data nested within persons were used to examine the effects of formal services on stress and depression. Results: For the GLMM procedure, this study used caregiving stressors and stress variables as fixed effects and participants as random effects. Results indicated that use of formal services significantly lowered caregivers’ stress and depression. Conclusion: The findings demonstrate the applicability of a daily diary approach and the effectiveness of formal services on the stress of Japanese family caregivers.

